# On the utilization of novel spectral laser scanning for three-dimensional classification of vegetation elements

**DOI:** 10.1098/rsfs.2017.0039

**Published:** 2018-02-16

**Authors:** Zhan Li, Michael Schaefer, Alan Strahler, Crystal Schaaf, David Jupp

**Affiliations:** 1 School for the Environment, University of Massachusetts Boston, 100 Morrissey Blvd., Boston, MA 02125, USA; 2 CSIRO Agriculture and Food, GPO Box 1700, Canberra, ACT 2601, Australia; 3 Australian Plant Phenomics Facility, The High Resolution Plant Phenomics Centre, CSIRO Agriculture and Food, GPO Box 1700, Canberra, ACT 2601, Australia; 4 Department of Earth and Environment, Boston University, 675 Commonwealth Ave., Boston, MA 02215, USA; 5 CSIRO Land and Water, GPO Box 1700, Canberra, ACT 2601, Australia

**Keywords:** dual-wavelength scanning, lidar, DWEL, vegetation structure, classification

## Abstract

The Dual-Wavelength Echidna Lidar (DWEL), a full waveform terrestrial laser scanner (TLS), has been used to scan a variety of forested and agricultural environments. From these scanning campaigns, we summarize the benefits and challenges given by DWEL's novel coaxial dual-wavelength scanning technology, particularly for the three-dimensional (3D) classification of vegetation elements. Simultaneous scanning at both 1064 nm and 1548 nm by DWEL instruments provides a new spectral dimension to TLS data that joins the 3D spatial dimension of lidar as an information source. Our point cloud classification algorithm explores the utilization of both spectral and spatial attributes of individual points from DWEL scans and highlights the strengths and weaknesses of each attribute domain. The spectral and spatial attributes for vegetation element classification each perform better in different parts of vegetation (canopy interior, fine branches, coarse trunks, etc.) and under different vegetation conditions (dead or live, leaf-on or leaf-off, water content, etc.). These environmental characteristics of vegetation, convolved with the lidar instrument specifications and lidar data quality, result in the actual capabilities of spectral and spatial attributes to classify vegetation elements in 3D space. The spectral and spatial information domains thus complement each other in the classification process. The joint use of both not only enhances the classification accuracy but also reduces its variance across the multiple vegetation types we have examined, highlighting the value of the DWEL as a new source of 3D spectral information. Wider deployment of the DWEL instruments is in practice currently held back by challenges in instrument development and the demands of data processing required by coaxial dual- or multi-wavelength scanning. But the simultaneous 3D acquisition of both spectral and spatial features, offered by new multispectral scanning instruments such as the DWEL, opens doors to study biophysical and biochemical properties of forested and agricultural ecosystems at more detailed scales.

## 1. Introduction

Light detection and ranging (lidar) is an active remote sensing technique using an instrument that emits coherent laser light to measure the distance to a target. The outgoing laser pulse interrogates targets along the transmission path, these targets induce scattering events, and light is scattered back towards the lidar instrument. In its simplest form, the lidar instrument records the travel time and intensity of the scattered light as it is received by its detector, allowing the distance to the target to be calculated. The travel time can be measured using either pulse ranging or continuous wave ranging techniques. Pulse ranging lidar is more commonly used for applications to vegetation [1], which is the subject of interest in this paper. Thus, we will confine our discussion here to pulsing lidar unless otherwise noted.

A recent application of lidar is the quantification of forest structure, and, in particular, measures of the physical dimensions of trees, the amount and location of leaves, and gaps between and within tree canopies, as well as secondary measurements such as tree volume. All of these measurements can be determined through the three-dimensional (3D) information acquired by lidar instruments on different platforms, including space-borne satellites, aircraft and on-ground mobile systems, as well as stationary terrestrial laser scanning (TLS) platforms.

In general, lidar return signals are recorded by two types of technologies, discrete-return and full-waveform. Discrete-return lidars record one to several laser returns as the laser beam passes through the canopy. Collecting multiple returns throughout a vegetation canopy is based on the intensity of the laser energy returned to the sensor [2]. By contrast, waveform lidars digitize the total amount of energy returned to the sensor at fixed time (and so distance) intervals, providing a continuous distribution of laser energy for each laser pulse [2]. In practice, this means that all scattering events, as well as the shape of the return pulses, within a plant canopy will be fully recorded for both full and partial-hit returns.

The focus of this paper is a full-waveform instrument, the Dual-Wavelength Echidna^®^ Lidar (DWEL) [3,4]. DWEL is a research instrument developed jointly by Boston University in the USA and CSIRO in Australia. It is a novel terrestrial lidar that uses two coaxial lasers at 1064 nm and 1548 nm wavelengths to collect simultaneous dual-wavelength scans. This unique design adds a new spectral domain to the TLS data by associating 3D ‘clouds’ of lidar points from scanned targets with their reflectance at the two wavelengths, which opens doors to 3D mapping of biophysical and biochemical properties of forests and other new applications. A few studies have demonstrated the value of this new spectral domain of lidar data for estimating vegetation biochemical properties [5,6]. A better interpretation and understanding of these forthcoming new 3D retrievals of vegetation properties presents the need to classify vegetation elements in 3D space for the differentiation of leaves, the primary photosynthetic component of vegetation, from the non-photosynthetic component, mainly woody materials. An advantage of DWEL scans for 3D classification is that the contrast of well-calibrated dual-wavelength lidar returns reduces or cancels out the effects of ranges, optics, electronics and vegetation structure on the return signals and thus provides a mechanism for the discrimination of different vegetation element types based on their instrinsic bispectral reflectance contrast [7].

Previous studies have addressed the separation of leaves from woody matter, by using ground-based optical remote sensing data for more accurate retrieval of leaf area index (LAI), foliage profiles and other structures. The woody-to-total area ratio derived from labour-intensive destructive sampling is widely used to correct the contribution of scattering by woody materials to LAI measurements by optical instruments [8]. Kucharik *et al*. [9] developed a multi-wavelength camera to separate leaves from woody materials to study the woody-to-total area ratio of a forest stand and improve the indirect measurements of LAI with optical instruments. These efforts to separate photosynthetic and non-photosynthetic materials remained in two-dimensional space with the aim to improve the LAI of a whole forest stand.

Several studies have also attempted the leaf-wood differentiation in 3D lidar point clouds of single wavelengths. Some early studies tried the manual extraction of trunks against leaves from lidar scans [8] and finer discrimination of leaves from both trunks and branches through the manual comparison between leaf-on and leaf-off scans [9]. Later studies attempted automatic 3D classification of the two components using point clouds from single-wavelength TLS operating at shortwave-infrared (SWIR) bands (1550 nm or similar) [10,11] or green band (532 nm) [12] by simply thresholding lidar return intensities. But the selection of intensity thresholds is subjective or needs adjustment from scan to scan. A recent study by Béland *et al*. [13] pushed the 3D point classification of leaves and woody materials further by developing a geometric-based automatic point classification algorithm using spatial distribution patterns of points for preliminary separation and a series of post-processing filters to achieve the final classifications.

Despite the long history of studies on separating leaves from woody materials, the 3D classification of these two vegetation components has only been possible because the application of TLS to forest studies and remains a challenging task. The primary aim of this paper is to demonstrate the strengths of the new spectral domain from our recently built novel dual-wavelength TLS for the 3D classification of vegetation elements. We used two nearly identical DWEL systems, the Oz-DWEL based in Australia and the National Science Foundation (NSF) DWEL based in the USA over six forest sites and an agricultural site. In this study, we examine the quality of spectral information from DWEL bispectral scans, compare its effectiveness for 3D classification with the traditional spatial information from lidar and present a synergy between spectral and spatial information to improve the 3D classification, paving the way for the study of biophysical and biochemical properties of forested and agricultural ecosystems at more detailed scales.

## 2. Material and methods

### 2.1. Study sites

To evaluate the bispectral information from DWEL scans and its application to 3D classification, we chose to test it on seven different locations that had been scanned using either of the two DWEL instruments. Each of these locations had a unique vegetation type, structure and form, varying from (table 1) temperate mixed forest in Massachusetts, USA, to tall eucalypt forests in Tasmania, Australia, through to vineyards in South Australia. Although these sites presented different challenges for scanning with the DWEL instruments due to factors such as access to the site, different vegetation types or the surrounding terrain, a standard scanning methodology was followed at each location.

**Table 1.** List of point classification test sites representing a variety of different vegetation ecosystems.

site location	forest type	scanning date	canopy condition	scanning instrument
Harvard Forest, MA, USA	temperate mixed deciduous and evergreen forest	20140503	leaf-off	NSF-DWEL
		20140919	leaf-on	NSF-DWEL
Karawatha Forest, QLD, Aus	Eucalyptus	20140924	evergreen	Oz-DWEL
Brisbane Forest Park, QLD, Aus	Eucalyptus	20140925	evergreen	Oz-DWEL
Tumbarumba Forest, NSW, Aus	temperate wet sclerophyll eucalypt forest	20140807	evergreen	Oz-DWEL
Warra Forest, TAS, Aus	tall eucalypt forest	20150204	evergreen	Oz-DWEL
National Canberra Arboretum, ACT, Aus	urban forest plantation	20150818	evergreen	Oz-DWEL
Nuriootpa Vineyard, SA, Aus	agriculture	20160324	mixed	Oz-DWEL

### 2.2. DWEL scanning details

The two DWEL instruments have been deployed in many different vegetation ecosystem types and environments in the USA and Australia. For this study, we scanned at the Harvard Forest during both the leaf-on and leaf-off conditions of the deciduous trees (table 1), while at the other locations forests are evergreen. The scanning protocol took the form of five scans, one located at the centre of the study plot, and four surrounding scans located around the centre at each of the cardinal points.

The DWEL is designed to operate in three different scanning resolutions of 1, 2 and 4 mrad, paired with laser beam divergences of 1.25, 2.5 and 5.0 mrad, respectively, using different sets of optical lenses to provide gapless coverages of the hemisphere [3,4]. The finer resolution scans need longer scanning time. To balance the scanning resolution and time, we have chosen 2 mrad scanning resolution with 2.5 mrad beam divergence for both instruments throughout all the scans used in this study. Once set up and running, each scan takes approximately 45 min to complete and the processed data results in a full dual-wavelength 3D representation of the environment surrounding the scan location. Some other key technical specifications of the DWEL instruments are given in table 2 and more details can be found in [3,4]. Of particular importance to and unique to the DWEL instruments among those specifications is the laser beam alignment error. Unlike most terrestrial lidars using only a single laser, the DWEL design requires two coaxial lasers to scan targets simultaneously. To ensure the two lasers interrogate the same target at the same time, it is important to minimize the coaxial laser alignment error to reduce artefacts in the contrast of lidar returns at the wavelengths of the two lasers. The current assessment of the NSF-DWEL instrument reported an alignment error of 0.79 ± 0.06 mrad when using the 2.5 mrad beam divergence. This alignment error translates to about approximately 60% overlap between the two laser beams and carries significant implications to the utilization of bispectral attributes of DWEL return signals which is further discussed in the §3.

**Table 2.** Key technical specifications of DWEL instruments [3,4].

	1064 nm	1548 nm
beam divergence (mrad)	1.25/2.5/5.0	
scanning resolution (mrad)	1.0/2.0/4.0	
outgoing pulse length (ns)	5.0 ± 0.1	
pulse repetition frequency (kHz)	2	
azimuth scanning range (°)	0–360	
zenith scanning range (°)	0–117	
laser beam alignment error^a^^,^^b^ (mrad)	0.79 ± 0.06	
single-target range resolution^a^ (1*σ*, cm)	4.752 ± 0.006	2.333 ± 0.001
leaf-like^c^ SNR^d^ at 100 m (70 m)^a^ (unitless)	4(10)	3(10)

^a^This specification is from the assessment of NSF-DWEL instrument using 2.5 mrad beam divergence.

^b^It was termed interimage alignment in [4].

^c^Leaf-like reflectance that was used in the assessment: 0.431 at 1064 nm and 0.239 at 1548 nm.

^d^Signal-to-noise ratio.

### 2.3. Bispectral point cloud production

Each individual DWEL scan generates a monospectral point cloud file for each of the two laser wavelengths, 1064 and 1548 nm. Before merging these two point clouds together to generate the bispectral (dual-wavelength) point cloud, we first convert the intensity values of points in digital numbers to apparent reflectance *ρ*_app_ (unitless), a physically defined quantity that is related to the radiative and structural characteristics of scanned targets and independent of range and instrument optics and electronics, using the radiometric calibration method in [7]. This radiometric calibration uses a semi-empirical model that combines a generalized logistic function to explicitly model telescopic effects due to defocusing of return signals at near range with a negative exponential function to model the fall-off of return intensity with range [7]. After generating *ρ*_app_ at both wavelengths respectively, we developed two ways of merging two DWEL monospectral point clouds: (i) the intersection approach and (ii) the union approach. The intersection approach finds common points in 1064 and 1548 nm point clouds that are matched with laser shot sequence number and ranges. To be specific, if two points have the same shot number and their range difference is smaller than a given threshold, then the two points are matched as returns from the same target. These points are saved to the bispectral point cloud with both 1064 and 1548 nm apparent reflectance values.

Besides the common points found by the above intersection approach, the union approach further takes into account the fact that points at one wavelength may not have corresponding points at the other wavelength. This mismatch can happen when a target has different reflectance values at the two wavelengths resulting in a low return signal, below the instrument's detectability at one wavelength, but not the other wavelength. The union approach synthesizes the *ρ*_app_ value of a point that is missing at a wavelength from the *ρ*_app_ value of the other laser wavelength with the help of a synthesized normalized difference index value (NDI, equation (2.1)) of the laser shot. The NDI value for this laser shot could be from average *ρ*_app_ values at the two wavelengths in the same shot or interpolated from neighbouring shots if this shot does not have any matched point pairs between the two wavelengths. In the following equation of the NDI calculation, the superscripts *nir* and *swir* denote the near-infrared (NIR) band of 1064 nm and the shortwave-infrared (SWIR) band of 1548 nm, respectively.2.1
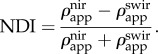
For the classification procedure developed in this paper, we use the union approach as it maximizes the number of points in the bispectral point cloud that are available for spectral and spatial classification.

### 2.4. Classification methods

The 3D classification algorithm exploits the spectral attributes of individual points from the DWEL scans in a supervised classifier, the random forest classifier (RFC) [14] implemented in an open source Python package Scikit-learn [15]. The spectral attributes of a point that our classification algorithm uses here include 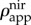 and 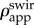, the two apparent reflectance values at both 1064 and 1548 nm, and the NDI value. We also apply the same classifier to spatial attributes of points as well as to the combination of spectral and spatial attributes to investigate their respective strengths and weaknesses for 3D classification. The spatial attributes of a point used here refer to the multiscale characteristics of the local 3D organization of a point and its neighbouring points within spheres of different diameters (scales). The 3D spatial organization of points in a local sphere, which we will call dimensionality here, is quantified by the proportion of each eigenvalue of the principal components of the recentred Cartesian coordinates of the points in this local sphere [16], and thus varies from 1D (points set along a line, one dominant eigenvalue and two diminutive eigenvalues), to 2D (points set on the surface of a plane, two similarly large eigenvalues and one diminutive eigenvalue) and 3D (points located freely in a 3D volume, three similar eigenvalues). The dimensionality may also change with the scale at which we view a cluster of points, and, therefore, the dimensionality metrics at multiple scales are included for the classification. We select the scales considering the minimum space between points given the scanning resolution for the minimum scale and the approximate distance between stems for the maximum scale.

**Figure 1.**
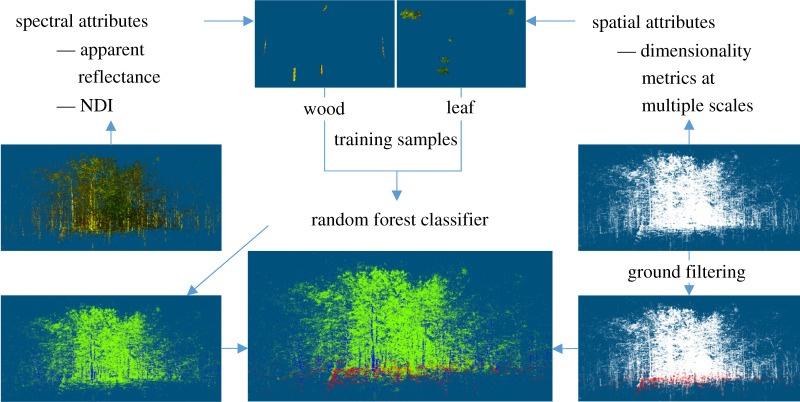
Diagram of the 3D classification method using the RFC and ground filtering algorithms.

To train the random RFC, we identify clusters of points as the training sets of woody and leaf matter using open source point cloud analysis software, such as CloudCompare or Meshlab, by visually inspecting the colour and shapes of point clusters that are rendered in composite colour with 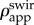 of a point as red, 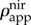 as green and a dark constant as blue. Selecting point clusters of pure classes, particularly pure leaves, is very difficult, if not impossible, as most leaves except those really close to the scanner are usually smaller than the laser spot sizes at the target ranges given our scanning resolution of 2 mrad. As a result, our training point samples inevitably include mislabelled points. The supervised classifier RFC, however, is known to be robust to noise in the training data thanks to its strategy of growing multiple classification trees [14]. These training points are used to train the RFC before it is applied to classify a whole point cloud. A separate ground point filtering process [17] is then performed on the point cloud to separate the ground from the rest of the ‘vegetation’ returns (figure 1).

The classification outputs of this algorithm are threefold in that we produce a *spectral* classification using only spectral attributes, a *spatial* one using only spatial attributes, and a *spectral–spatial* one using both types of attributes. The generation and comparison of the three outputs identifies the strengths and weaknesses of spectral and spatial attributes on 3D classification of vegetation elements and elucidates the causes of their qualities, especially the new bispectral attributes.

## 3. Results and discussion

### 3.1. Strengths of spectral and spatial attributes from classification comparison

**Figure 2.**
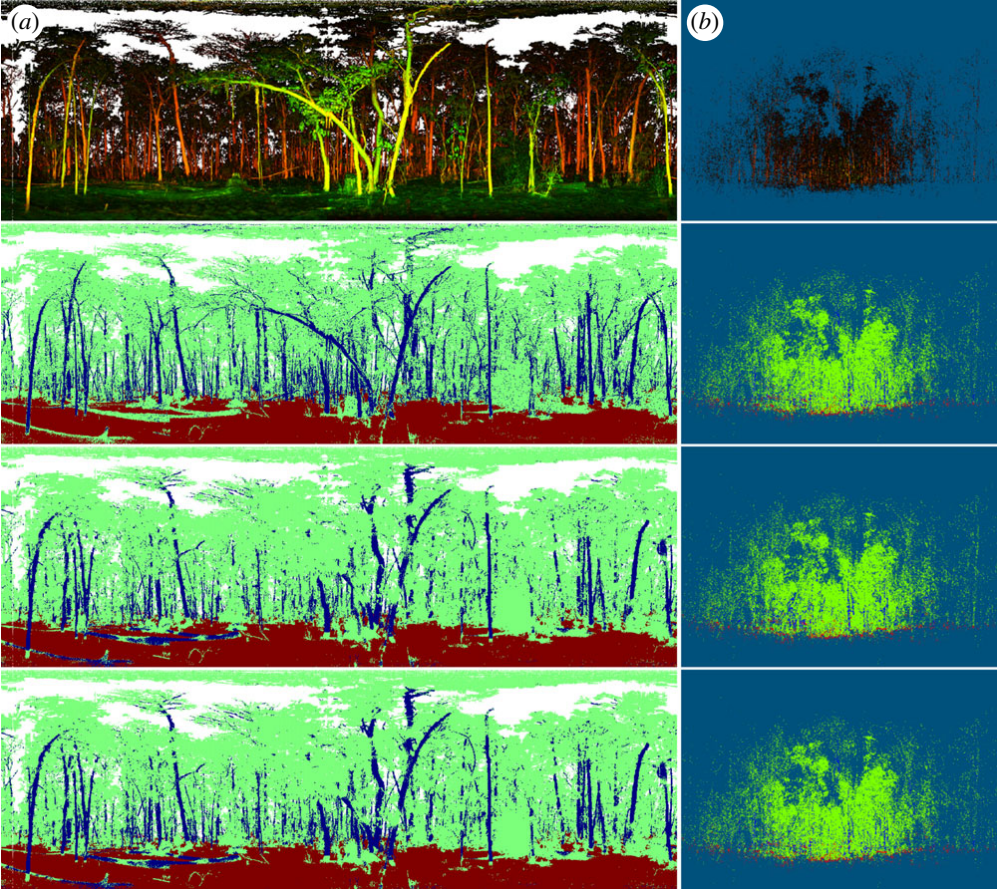
Overview of the classification of one example scan at Tumbarumba Forest, NSW, Australia. From the first row to the fourth: (1) colour-composite images, (2) spectral classification, (3) spatial classification and (4) spectral–spatial classification. (*a*) Equal-angle projection image and (*b*) is the point cloud. In the classification images, colours represent different elements; red, ground; green, leaves; blue, woody matter. The white areas in the equal-angle projection images are gaps without returns.

The comparison between the three point classification results, i.e. spectral, spatial and spectral–spatial, reveals that the spectral and spatial attributes generally complement each other's strengths (although not always), in differentiating leaves and woody matter in the 3D space. Figure 2 displays the overview of the colour-composite scanning image and the three classification results of the centre scan at Tumbarumba Forest site, NSW, Australia, as an example (from top row to bottom as the colour-composite scan and the spectral, spatial and spectral–spatial classifications). It is difficult to visually see the details of a whole 3D point cloud in still pictures. Therefore, we project the 3D classified points in the right column of figure 2 to a 2D equal-angle projection in the left column where the *X*-axis of the projection is azimuth and the *Y*-axis is zenith for a better display. However, it is stressed that our classifications of vegetation elements are carried out in 3D space rather than the 2D. From the overview of the projection images, overall the spectral classification resolves more fine branches, especially those inside the upper canopy, than the spatial classification. Conversely, the spatial classification method produces cleaner identification of trunk points, which is especially evident at the trunk edges. Additionally, the spatial classification method helps classify targets with unusual spectral responses, particularly trunks and branches with atypically high water content.

The spectral and spatial attributes for vegetation element classification are suitable for different parts of vegetation (canopy interior, fine branches, coarse trunks, etc.) and under different vegetation conditions (dead or live, leaf-on or leaf-off, water content, etc.). These environmental characteristics of vegetation are convolved with lidar instrument specifications and lidar data quality, which results in the actual capabilities of spectral and spatial attributes to classify vegetation elements in 3D space. The point classification results from our seven test sites suggest the following typical cases where the application of either spectral or spatial attributes performs better than the other, while using them together could improve the classification. Generally the spectral attributes are better at the classification of leaves and woody matter for the following cases.

**Figure 3.**
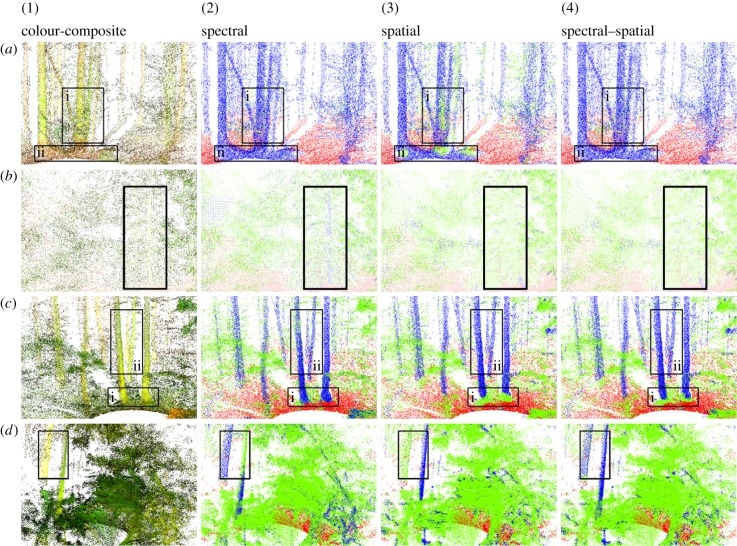
Zoom-in images of point clouds from the left column to the right in composite colour and spectral, spatial, and spectral–spatial classification results for different scans at (*a*) Harvard Forest north scan, leaf-off; (*b*) National Canberra Arboretum; (*c*) Harvard Forest north scan, leaf-on; (*d*) Warra Forest. In the classified point clouds, colours represent different elements; red = ground, green = leaves and blue = woody matter. The black boxes highlight the difference between the three classifications as described in the §3.1.

(1) *Clusters of trunks and branches*. Points in such clusters will display the dimensionality metric of near 3D volume shape that mostly indicates leaf points according to our training samples. Hence spatial classification is likely to fail while the spectral attributes differentiate these woody points from leaf points, e.g. figure 3*a* (box i), noticing the misclassified leaf patch (green) on the trunk (blue) in the spatial classification but correctly identified by spectral and spectral–spatial classifications.
(2) *Intersection between trunks and ground*. The spatial attributes suffer the same problem with classifying the points of these cases as the last case of cluster trunks and branches, e.g. figure 3*a* (box ii) and figure 3*c* (box i), noticing the larger leaf patch (green) in the spatial classification neary the trunk bases than in the spectral and spectral–spatial classifications.
(3) *Interior of canopy*. Point clusters inside canopy generally mix returns from both leaves and woody matter together and thus bear similar multiscale dimensionality metrics. Therefore, it is difficult for the spatial classification to work well for interiors of canopies, e.g. as to identify a stem inside the dense canopy of an evergreen tree as shown in figure 3*b*. However, noticing the thin blue dots highlighted by the black box in spectral classification (second column), it is the stem captured by its distinct spectral attributes than surrounding leaves.

On the other hand, the spatial attributes are better at the classification generally for the following cases.

(1) *Unusual spectral response of vegetation elements*. The stronger spectral contrast of leaves than woody matter at the two wavelength of DWEL lasers has been observed by spectroscopic measurements of various forest objects [18] and our early pilot DWEL scans [3]. However, we have discovered outliers, particularly some peculiar woody matter from our scanning campaigns. Some woody matter shows much lower reflectance at the SWIR band than the NIR just as leaves do, for example, the right greenish trunk marked by box ii of figure 3*d* (first column). This greenish trunk appears to have higher water content than usual. Indeed, it is known that some tree stems do have photosynthetic capacity with living cells at the surface [19,20]. The lidar returns of these woody matter with leaf-like spectral reflectance are labelled incorrectly by spectral classification (figure 3*d*, second column). These spectrally peculiar woody materials, however, are identified correctly by their spatial attributes (figure 3*d*, third column).
(2) *Trunk edges*. The misclassification of trunk edges by spectral attributes results from the misalignment of the two lasers at the two wavelengths that causes the two lasers to illuminate different spots on the trunks. This mismatch between two illuminated spots may not matter for the classification as long as the two spots cover similar trunk surface. However, at trunk edges where laser beams may be only partially intercepted, the area intercepted by one laser can differ in size from the other if the two lasers are not aligned. The contrast in the bispectral attributes for such cases is not caused by the instrinsic spectral features of woody matter but artefact from the laser misalignment. As a result, we note these misclassification of trunk edges by the spectral classifications (figure 3*c*, second column, box ii, but hard to see them without using point cloud visualization softwares because they are usually narrow strips of points along trunks in the point cloud). The spatial attributes do not suffer from this laser misalignment and thus helps improve the classification of trunk edges in such cases.

Furthermore, there are a few cases where neither spectral nor spatial attributes yield a robust classification or using them together does not always improve the result.

(1) *Targets at far ranges*. The uncertainty in the apparent reflectance, i.e. spectral attributes, may be quite large at far ranges due to a low signal-to-noise ratio of weak returns [7]. This results in poor quality of spectral attributes in terms of *ρ*_app_ at each wavelength and the spectral contrast between wavelengths. Meanwhile, targets at far ranges return low-density points, which induce considerable uncertainty in the calculation of multiscale dimensionality metrics. The resulting low quality of spatial attributes then misclassifies these targets.
(2) *Compounds of the above cases*. The natural environment can be complicated and compounding of the above cases is not uncommon. Under such circumstances, the strengths of spectral or spatial attributes may be compromised by strong weaknesses of the other when using both types of attributes in the classification.

The above examination of the strengths and weaknesses of spectral and spatial attributes primarily concerns the quality of the identification of woody material. After all, it is easier to visually discern pure woody matter such as trunks and branches in scans (either projected images or as point clouds) for the classification assessments, due to their distinctive shapes and relatively large sizes as compared to laser beam spots even at far ranges. In contrast, identifying pure leaf points by visual examination is much more difficult. However, our classification simply discriminates between two classes—leaves and woody matter. This binary classification scheme implies a better identification of one class (woody matter), which to some extent also indicates a better identification of the other class (leaves). Therefore, the examination of the classification results here is not completely infallible, but still informative for the classification performances of both woody matter and leaves.

The examination of the classification results is qualitative due to the lack of ground truth datasets in 3D space. A quantitative assessment of point classification accuracy is certainly needed, but generally calls for an independent data source of 3D truth labels of each point. It is difficult to obtain 3D pointwise ground truth data independent of lidar scans. Destructive sampling, while providing the overall volume or weight of leaves versus woody materials, is extremely difficult and rarely performed. While 2D photos taken by some lidar instruments in tandem with scanning can aid classification assessments, not every point can be registered to available photo pixels, especially for multiple returns along laser beams. Therefore, using such 2D photography for 3D classification accuracy assessement needs further development. The common practice to acquire ground truth data for 2D remote sensing image classification relies on reference data of higher quality than the data used for classification, or if using the same source, requires a more accurate process of creating the reference classification than the classification to be assessed [21]. Such practices cannot be direclty transferred to the 3D point classification assessment, and new approaches and techniques need to be developed for the quantitative accuracy asssessment of 3D point classification.

**Figure 4.**
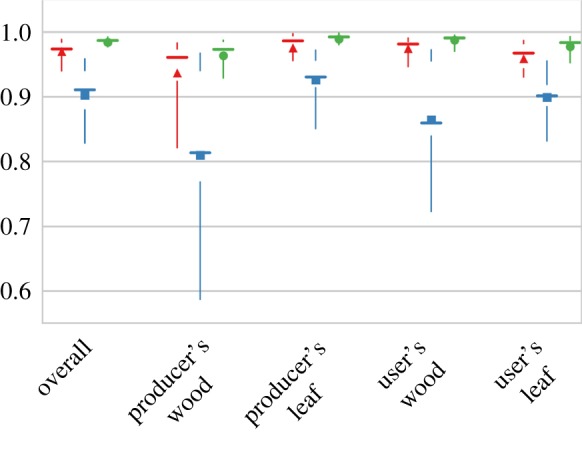
Boxplot of the overall, producer's and user's accuracies of the three classifications (red triangle, spatial; blue square, spectral; green dot, spectral–spatial) on all the scans from the cross validation. The triangles/squares/dots are mean values of cross-validation accuracies and the short horizontal lines are median values. The gaps between two whisks (vertical lines) are the quartiles and the extent of the whisks are 5 and 95 percentiles. (Online version in colour.)

Nonetheless, we compared the performances of the three classifications based on the training data using a cross-validation technique. We took 75% of the points from the training data of a scan using stratified random sampling to train the RFC and used the remaining 25% points with known labels to validate the classification result and calculate the overall accuracy. As noted earlier in the §2.4, our training data inevitably includes mislabelled points and thus the overall accuracy obtained in this way is flawed. However, we can assess the relative performance of the three classifications by comparing the mean and median of the overall, producer's, and user's accuracies of all the scans from this cross validation (figure 4). The spectral–spatial classification has the highest cross-validation overall accuracy, followed by the spatial classification and then the spectral one, although all the three report more than 90% overall accuracies, and are thus on a par with each other. But using both spectral and spatial attributes for the classification, the variance of the accuracies across all the scans is signficantly reduced, endorsing the important values of both atttributes for a robust classification across different vegetative environments. The producer's accuracy (or omission error) and user's accuracy (or commission error) also demonstrate the reduction of accuracy variance from the synergy between these two types of attributes. The effectiveness of and sources of uncertainty from the spectral and spatial attributes result in the different performances provided by the spectral and spatial classifications, which are discussed in the next section.

### 3.2. Effectiveness and sources of uncertainty of spectral and spatial attributes

**Figure 5.**
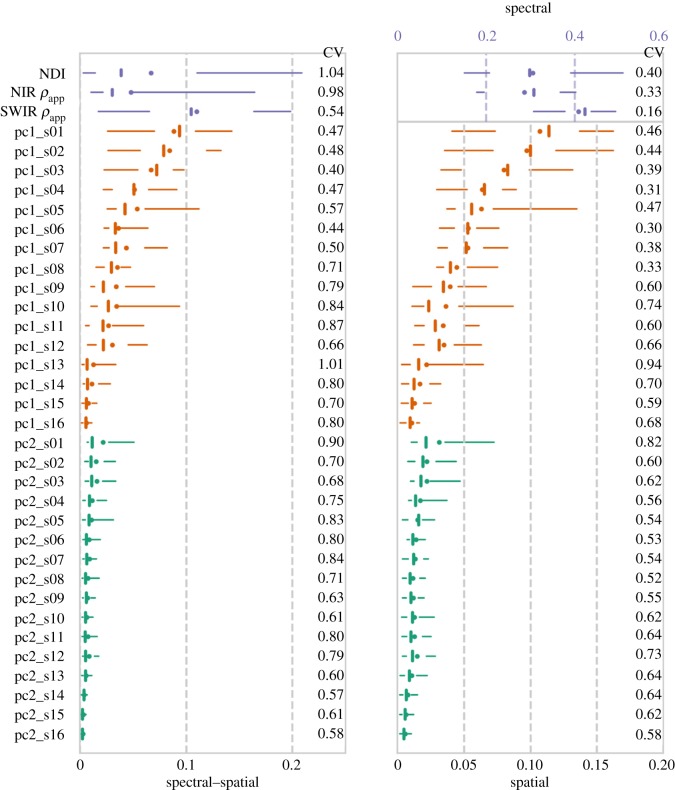
Boxplot of feature importance to RFC from all the scans in the three classification types: spectral–spatial, left panel; spectral, upper right panel; spatial, lower right panel. Each line is for a feature from spectral or spatial attributes. The purple represents spectral attributes. The orange and the green represent the spatial attributes at different scales, where pc1 and pc2 are the proportions of the first and second eigenvalues from the PCA on the recentred Cartesian coordinates of a local point cluster (see §2.4 for more details). The suffix from ‘s01’ to ‘s16’ represent the scales at which the spatial attributes are calculated, from 1 m (s01) to 0.05 m (s16). The dots are mean FIS and the short vertical lines are median values. The gaps between two whisks (horizontal lines) are the quartiles and the extent of the whisks are 5 and 95 percentiles. The numbers on the right side of each panel are the CV of importance scores. (Online version in colour.)

Both spectral and spatial attributes contribute to the 3D classification according to their feature importance scores (FIS) from the RFC in the three classification types, using spectral attributes only (figure 5, upper right panel), using spatial attributes only (figure 5, lower right panel) and jointly using both types of attributes (figure 5, left panel). For the spectral attributes, the 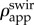 ranks the most important, followed by the NDI and the 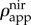 the least important, for both spectral classification and spectral–spatial classification. Leaves and woody matter share similar reflectance at the NIR band and thus the 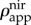 has the lowest FIS in the RFC. However, the lower FIS of the NDI than the 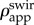 is not expected. The NDI, in theory, characterizes the intrinsic spectral difference between leaves and woody materials better than the 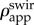 because the dual-wavelength-based NDI cancels out the effect of vegetation structural characteristics on the apparent reflectance, while the single-wavelength 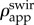 does not. In this case, the misalignment of the two laser beams may reduce the fidelity of the bispectral difference signals from the NDI. The unexpected FIS of the NDI, lower than the 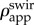, suggests that the laser misalignment may be affecting the quality of spectral information from dual-wavelength scans. The current laser beam alignment error of 0.79 ± 0.06 mrad using 2.5 mrad beam divergence (table 2) results only in 60% overlap of different laser beam spots between the two wavelengths [4]. As a worst case example, for a leaf cluster with reflectance of 0.431 at 1064 and 0.239 at 1548 nm (table 2), this spot difference could alter the true NDI of 0.287 to 0.154, an error of almost 50%, assuming that 40% (the non-overlap part) of the 1548 nm laser intercepts branches with reflectance 0.431 at both wavelengths but the 1064 nm all intercepts leaves.

The spatial attributes, presented in figure 5 as (pc1_s01-pc1_s16) and (pc2_s01-pc2_s16), are the proportions of the first and second eigenvalues from the PCA on the recentred Cartesian coordinates of a local point cluster (see §2.4 for more details) at the 16 scales from 1 m (s01) to 0.05 m (s16). The larger ‘pc1’ at a given scale, the more likely a point cluster at this scale forms a 1D line shape. Similarly, the larger ‘pc2’, the more likely a point cluster forms a 2D plane shape at a scale. The FIS of the spatial attributes are not dominated by any single scale but similar between several scales (at least scale _1 to _3 in figure 5), which confirms the need of multiple scales to capture the spatial features of objects from point clouds. The proportions of the first eigenvalues from PCA (pc1 in figure 5) at larger scales are more important. When viewed at larger scales, the most distinct difference between leaves and woody materials is likely due to the 1D line shapes of trunks and branches versus the 3D volume shapes of leaf clusters. Therefore, the pc1, indicating the similarity to a 1D line shape, dominate the contribution to the classifications. At smaller scales, the point density is probably insufficient for the PCA to capture the spatial shapes of objects. Finer beam divergence can increase point densities at far ranges but imposes more demanding requirements of laser coalignment accuracy (see §3.3).

Comparing FIS distributions between the spectral and spatial classifications (right two panels in figure 5), the spectral attributes show smaller relative variations (coefficient of variation, CV) in FIS than the spatial attributes. The spectral domain of the DWEL scans carries the intrinsic spectral contrast of vegetation elements and thus contributes to the classification more consistently while varying spatial arrangements and sizes of vegetation elements across sites result in different effectiveness of spatial attributes to the classification. This site variation in the spatial attributes also explains the larger CV of FIS of spectral attributes in the spectral–spatial classification than in the spectral classification. Different effectiveness of spatial attributes to the classification across sites induces assimilating different levels of additional spectral contribution to the synergistic spectral–spatial classification. Comparing within the spectral–spatial classification, while the mean and median FIS of the spectral attributes are similar to the spatial attributes at several scales, the larger upper percentiles of spectral FIS suggest the great potential of spectral attributes in 3D classification. However, warranting this potential needs further improvements of spectral lidar manufacturing and data processing for better robustness in the quality of spectral attributes.

### 3.3. Potential improvements in utilization of spectral and spatial information

The aforementioned examination and analysis of our classification results reveal the quality issues associated with both spectral and spatial attributes and offer several potential improvements in the utilization of the spectral and spatial information from the novel spectral TLS for forest ecology, such as DWEL. First, the efficacy of spectral attributes relies on the accuracy of both the radiometric calibration and laser beam alignment. A better radiometric calibration gives more accurate apparent reflectance at each wavelength, i.e. the individual spectral attributes. Points at far ranges nonetheless will have less accurate apparent reflectance values, due to the fall off of SNR with range. It is possible to take into account this uncertainty of spectral attributes for a better spectral–spatial classification or other applications, by, for example, using smaller weights on spectral attributes for points at far ranges. The quality of spectral information also requires an accurate laser beam coalignment to minimize artefacts in the spectral contrast and provide better presentations of instrinsic spectral features of vegetation elements in 3D space. Analogous to multispectral imaging sensors, we cannot really use the spectral bands unless these bands are well registered in order to ensure the pixels from all the bands represents the same target. Although a single supercontinuum laser (while laser) has been prototyped for indoor laboratory test of a spectral scanning lidar to avoid multilaser coalignment [22], there seems a much longer path to achieve its operational scanning due to the high cost of white lasers and technical difficulties of balancing the safety of white laser powers and detectabilty of splitted return energy at each wavelength.

Second, for the spatial attributes, a good selection of scales is important, but there are no good scales universal for every part of a scan or different sites. It is possible to select a set of reasonable scales for most parts of a scan according to scanning resolutions and general distances between tree trunks. However, several common cases, such as varying point densities with ranges and branch sizes, clusters of trunks and/or branches, etc., call for different scales to better capture the spatial shapes of targets for classification. Merging multiple scans via registration will alleviate the impact of varying point density on the quality of spatial attributes, but not completely. Separating individual trunks and branches from a cluster may improve the quality of spatial attributes by calculating the dimensionality metrics using points separately from individual trunks and branches. Therefore, point cloud segmentation algorithms (such as the covering technique to partition points into small clusters in the quantitative structure model [23]) can be applied to generate 3D primitives or patches for object-based classification. Higher point density from finer beam divergence and scanning resolution (such as those currently offered by commercial single-wavelength TLS) can also help resolve the spatial shapes of vegetation elements of small sizes or at far ranges. But for spectral lidars, this finer beam divergence imposes even more demanding laser coalignment accuracies for the application of new spectral information to 3D mapping of biochemical and biophysical properties in vegetative environments. Before achieving a low-cost building of such high-quality and rugged spectral lidars for large-scale outdoor forest scans, good balance between beam divergence and laser coalignment accuracy needs to be identified for specific applications and the combination of single-wavelength lidars of very fine beam divergence and dual-/multi-wavelength lidars can be explored.

A further fine-tuning of the classification can also be performed using the full-waveform nature of the scans. Full-waveform data retains return pulse shapes that prove helpful to point cloud classification. Tree trunks that intercept laser beams near-orthogonally return single sharp pulses, while soft vegetative targets such as leaf clusters, or oblique surfaces such as trunk edges, return single/multiple elongated pulses [24]. The contrast in return pulse widths between tree trunks and leaf clusters can aid the classification by examining the ratio of pulse peak intensity to pulse width but still needs spectral and spatial attributes to reduce the confusion between leaf clusters and trunk edges [24].

## 4. Conclusion

This study of vegetation element classification in 3D space explores the values of spectral and spatial inforation simulataneously offered by the novel dual-wavelength lidar, the DWEL. The analysis of three different classifications highlights the spectral and spatial attributes that each perform better at identifying different parts of vegetation (canopy interior, fine branches, coarse trunks, etc.) under different vegetation conditions (dead or live, leaf-on or leaf-off, water content, etc.). These environmental characteristics of vegetation, convolved with the lidar instrument specifications and lidar data quality, result in the varying capabilities of spectral and spatial attributes to classify vegetation elements in 3D space.

We have observed that both spectral and spatial features of the point cloud contribute to the point classification and complement each other to provide a more robust and accurate classification result. Dual-wavelength laser scanning allows the use of the differential response at the two wavelengths, which offers a presentation of intrinsic spectral contrast of vegetation elements by reducing the range, telescopic and electronic effects as well as the vegetative structural influences on interpreting lidar return signals. The bispectral response thus provides an important new source of information that is potentially superior to single-wavelength scanning and allows more utilizations of 3D scanning data. At the same time, it introduces new sources of error that may be difficult to overcome, especially in a first-generation instrument. Better control of alignment, coupled with optimization of other scan parameters, such as beam divergence and angular sampling, will bring lower variance and higher accuracy to the dual-wavelength information.

Regarding the utilization of spatial attributes for the classification, multiple spatial scales are needed to capture well the spatial features of objects from point clouds. The selection of those scales is important, but it may be difficult to reach a consensus for different parts of a point cloud or different sites due to the complexity of forest environments and varying point densities. For example, at all but the finest scales, fine branch hits are likely to be misclassified when surrounded by leaves. To better capture spatial features, object-based spatial attributes and consequent classifications rather than point-based need to be investigated in the future by segmenting the point cloud into 3D primitives or patches. Finer beam divergence and scanning resolution can also help resolve the spatial shapes of vegetation elements of small sizes or at far ranges, however, for the applications of spectral lidars, on the condition of achieving more demanding laser coalignment accuracies imposed by finer beam divergence.

In our examples, a simple cross-validation test using the training data shows the value of including both spectral and spatial attributes in the classification, with the highest accuracies observed for spectral–spatial classification, followed closely by classifications using spatial and spectral attributes alone. However, spatial structure is more irregular across sites and hence contributes to the classification differently, while the bispectral domain offers an intrinsic feature of vegetation elements, the spectral contrast in their reflectance that contributes to the classification more consistently across sites. This ability not only enhances the accuracy of the spectral–spatial classification but also reduces its variance across the multiple vegetation types we have examined.

In conclusion, the value of dual-wavelength spectral scanning for the separation of leaf and woody signals is enhanced by simultaneously utilizing 3D spectral and spatial data. Together, the two information domains acquired by the DWEL instrument provide a new model that shows great promise for full exploitation of bispectral information in studying vegetative environments.

## Data accessibility

All the bispectral DWEL point cloud data (1.5 GB after compression) used in this study can be downloaded at ftp://rsftp.eeos.umb.edu/data02/OpenAccessPaperData/RSFS-2017-DWEL.tar.gz. All the processing codes can be obtained from the authors on request.
